# Supplementary material for: Epidemiological, clinical, and geographical characterization of Leprosy in the County of Santarém-Pará: Insights for effective control and targeted intervention
Source: PLoS Negl Trop Dis. 2024 Mar 20;18(3):e0012063. doi: 10.1371/journal.pntd.0012063 (PMC10984465; doi:10.1371/journal.pntd.0012063)
Supplement: Attachment — Submitted filename: Dear Reviewers PLOS.pdf [file pntd.0012063.s001.pdf]

Dear Reviewers,

We sincerely appreciate the thorough review you conducted on our manuscript submitted to PLOS Neglected Tropical Diseases. Your insightful feedback played a pivotal role in enhancing the overall quality and clarity of our work. We carefully considered all your comments and suggestions, and we are pleased to inform you of the revisions implemented to address your concerns.

Response to Reviewer #1:

We acknowledge and appreciate your feedback regarding the originality of the paper. In response, we have revisited the manuscript, aiming to enhance its contribution to the field of leprosy epidemiology. To address your concerns, we have expanded the depth of our discussion, emphasizing the implications of our findings for leprosy control and public health strategies in the region.

Response to Reviewer #2:

Thank you for your comprehensive comments on various aspects of our article. To address your concerns, we made the following changes:

Racial Terminology: Revised the terminology from "Race" to "Self-Declared Skin Color" in Table 1 and throughout the manuscript to better capture the complexity of racial and ethnic identities in the Brazilian population.

Leprosy Form Description: Clarified the terminology used to describe forms of leprosy, aligning it with the Ridley-Jopling classification for improved clarity among a broader audience.

Spatial Mapping: Recognizing the significance of mapping in identifying leprosy outbreaks, we conducted a relative risk analysis by neighborhood in the urban area of the municipality of Santarém and included cluster analysis.

Response to Reviewer #3:

We appreciate your comments on our article and have incorporated the following revisions:

Spatial Analysis: Acknowledging the importance of spatial analysis, we added a map featuring relative risk analysis.

Data Presentation: To adhere to conventional formatting, we separated the Results and Discussion sections, presenting results first and then interpreting and discussing them.

Terminology and References: Addressed issues related to terminology and references in the manuscript to enhance clarity and accuracy.

We believe these revisions significantly enhance the quality and clarity of our manuscript, positioning it as a valuable contribution to the field of leprosy epidemiology. We are confident that these changes address your concerns and invite your feedback on these revisions. We hope the updated manuscript aligns with the publication standards of PLOS Neglected Tropical Diseases.

Thank you for your time and expertise.

Best regards,

Livia de Aguiar Valentim
