# Supplementary material for: Epidemiological, clinical, and geographical characterization of Leprosy in the County of Santarém-Pará: Insights for effective control and targeted intervention
Source: PLoS Negl Trop Dis. 2024 Mar 20;18(3):e0012063. doi: 10.1371/journal.pntd.0012063 (PMC10984465; doi:10.1371/journal.pntd.0012063)
Supplement: Attachment — Submitted filename: RESPONSE TO REVIEWERS PLOS.pdf [file pntd.0012063.s002.pdf]

Dear Reviewers,

We sincerely appreciate the thorough review you conducted on our manuscript submitted to PLOS Neglected Tropical Diseases. Your insightful feedback played a pivotal role in enhancing the overall quality and clarity of our work. We carefully considered all your comments and suggestions, and we are pleased to inform you of the revisions implemented to address your concerns.

RESPONSE TO **REVIEWER 1:**

The study lacks original insights: We emphasize that while the study focuses on the retrospective description of leprosy cases in a specific area of Brazil over a 10-year period, it provides a detailed insight into the epidemiological, clinical, and geographical situation, contributing to the understanding of the disease in that region.

Discrepancy in prevalence data: We appreciate the observation. We have corrected the prevalence data based on the numbers provided by the WHO, adjusting the rates to accurately reflect the situation in 2019, as suggested.

Lack of an analysis plan beyond the description of recorded cases: We appreciate the observation. We have introduced a more robust analysis plan that includes detailed analyses of racial and geographical variations in leprosy incidence. These points are now discussed more thoroughly in the manuscript.

Racial and geographical variations were not analyzed in detail: We acknowledge this gap. We have expanded the analysis to include a more in-depth discussion of racial and geographical variations in leprosy incidence, providing a more comprehensive and interesting approach.

Lack of original discussion points or conclusions: We appreciate the observation. We have reviewed the discussion and conclusions, introducing more original points and emphasizing practical implications of the results to enhance the study's contribution.

RESPONSE TO **REVIEWER 2:**

Appropriate study objectives and design: Thank you for the acknowledgment. We confirm that the objectives were clearly outlined, and the study design was planned to meet these objectives.

Adequate description of the population and sample size: We are pleased that the description of the population and the sample size were deemed appropriate. We have worked to ensure clarity on these aspects in the manuscript.

Appropriate statistical analyses: Thank you for the positive assessment of the statistical analyses. We confirm that the analyses were conducted appropriately to support the conclusions drawn in the study.

Absence of ethical or regulatory concerns: We confirm that we followed all ethical and regulatory guidelines during the development of the study.

The presented analysis aligns with the analysis plan: Thank you for the acknowledgment. We have implemented the suggested changes regarding leprosy forms, clarifying the distinction between Ridley-Jopling and forms peculiar to Brazil. Additionally, we have restored the original Portuguese names of some Basic Health Units translated into English to facilitate location.

The conclusions are mainly supported by the data: Thank you for the acknowledgment. We have strengthened the connection between the data and conclusions, emphasizing how the distribution of leprosy cases in the 48 neighborhoods can be used to identify hot spots and assist health officials in directing efforts to specific areas in leprosy control.

Table 1: Replacement of "Raça" with "Cor da pele" and correction of discrepant values: We appreciate the observation. We have corrected the table as suggested, replacing "Raça" with "Cor da pele" and adjusting the discrepant values, ensuring clarity and accuracy.

Line 179: Replacement of percentage with number of cases: We agree with the suggestion. We have replaced "percentage" with "number of cases" for greater precision, indicating 74 cases in 2014 and 20 cases in 2020.

Lines 184-186: Considerations about cases in children: We appreciate the observation. We have included suggested references to support observations about cases in children, improving the scientific foundation of this part of the study.

Table 2: Clarification of "ign/white" and correction of terms: We appreciate the observation. We have clarified the meaning of "ign/white" and corrected the terms as suggested to improve the understanding of the table.

Lines 227-228, 232, 238: Clarification of terms: We acknowledge the lack of clarity. We have added explanations about "tipo HD," "forma HD," and "HI" to ensure proper understanding.

Lines 243-244: Correction of the sentence about nerve lesions: We appreciate the suggested correction. We have modified the sentence to indicate that only 37 (6%) had more than 4 nerves affected.

Lines 251-261: Keep Brazilian names in Table 3: We appreciate the suggestion. We have retained the Brazilian names of neighborhoods and Basic Health Units (UBS) in Table 3 to avoid confusion and facilitate identification.

Race vs. Self-Declared Skin Color: We appreciate the suggestion. We have changed "Race" to "Self-Declared Skin Color" in Table 1 and in the Conclusion, considering the complexity of racial mixture in the studied population.

Description of Leprosy Forms: We value the observation. We have clarified the description of leprosy forms, avoiding the mixing of Brazil-specific terms and adopting a more consistent approach with Ridley-Jopling forms.

New Case Detection Rate (TNCD): We did not include them; instead, we chose to incorporate other analyses, such as cluster analysis and a map depicting the relationship between variables.

Geographic Information System (GIS): We appreciate the recommendation. Recognizing the importance of GIS mapping for spatial analysis, we have added a section to the manuscript highlighting the GIS location of the 581 cases on the map, offering a clearer and targeted visualization of case distribution in Santarém's neighborhoods.

RESPONSE TO **REVIEWER 3**:

Discrepancy between methods and results: We acknowledge the observation and have carefully reviewed the methods and results to ensure a clearer correlation between them. The sections have been adjusted to accurately reflect what was implemented and the results obtained.

No, the results are presented clearly. However, the mix of results and discussion is confusing: We understand the concern. We have reorganized the manuscript to clearly separate results from the discussion, following the standard format, allowing for a more seamless and understandable reading experience.
No, the tables would need considerable revision: We acknowledge this. Extensive revisions have been made to the tables to enhance clarity and data presentation.

Line 150: It is not clear why Results and Discussion are mixed: We appreciate the observation. We have corrected this inconsistency by following the normal sequence of presenting results separately before interpreting and contextualizing them in the Discussion, providing a more logical organization to the manuscript.

No clear conclusions, just a description of the distribution of leprosy cases in the study period: We appreciate the feedback. We have revised the conclusions, providing a clearer interpretation of the results. We highlight the practical relevance of the distribution of cases and how it can guide control strategies.

Lines 327-329: It seems that spatial analysis is missing; this would have been the most salient feature of this paper: We acknowledge this gap. We have included a more robust spatial analysis, emphasizing its importance and how it significantly enhances the understanding of leprosy distribution in the studied area, addressing the raised concern.

Line 32: We appreciate the observation and agree with the suggestion. We have changed the term to "municipality" to better reflect the geographic structure of Santarém-Pará.

Line 36: We agree with the observation. We have corrected the term "prevalence" to indicate the cumulative incidence of new cases detected during the study period.

Line 38: We have corrected the term "prevalence" to "cumulative incidence" in accordance with the observation.

Introduction: We have updated the information on prevalence in line with the new technical guidelines from the WHO on the interruption of transmission and elimination of leprosy (2023).

Results: We have separated the results from the discussion to follow the standard structure. In Table 1, we clarified that the p-values refer to the comparison of percentages. However, we agree with the suggestion to omit p-values and present percentages in a separate line for greater clarity. We removed the reference to Basso and adjusted explanations about prevalence in adults and children, considering the new guidelines from the WHO. We clarified the sentence about increased transmission, making it clearer and evidence-based. We adjusted the numbers of child cases from 2009 to 2014, as suggested. We changed "prevalence" to "predominance" in Line 201 and discussed possible reasons for differences in cumulative incidences between areas. We corrected the incorrect sections in Lines 205-2012. In Table 2, we clarified "ign/white" and "Informed 0-99" and agreed with the suggestion to omit p-values. We clarified that all cited studies are population-based. We adjusted Lines 240-241 to better reflect the reasons for the high proportion of MB.

Conclusion: We have reviewed the conclusion, making the conclusions clearer and adding a more robust spatial analysis

Nature of the Article: We acknowledge the observation about the article's nature. We emphasize the description of leprosy case distribution in Santarém, underscoring the practical utility of this information for prevention and control strategies.

Spatial Analyses: We understand the concern about spatial analyses. We have included the results of spatial analyses in the findings, providing more clarity on the salient features of the study.

Utilization of Data for Strategy Formulation: We reinforce the connection between the specific data presented and their utility in formulating effective prevention, control, and intervention strategies. We highlight how detailed information can guide targeted actions in the specific region of Santarém.
