# Supplementary material for: Epidemiological, clinical, and geographical characterization of Leprosy in the County of Santarém-Pará: Insights for effective control and targeted intervention
Source: PLoS Negl Trop Dis. 2024 Mar 20;18(3):e0012063. doi: 10.1371/journal.pntd.0012063 (PMC10984465; doi:10.1371/journal.pntd.0012063)
Supplement: Attachment — Submitted filename: Response reviewers_L.pdf [file pntd.0012063.s005.pdf]

Dear reviewers,
We sincerely appreciate all the considerations and suggestions on our manuscript. We believe we have answered and addressed all the questions presented and we believe that with this version it is possible to publish. I apologize for having missed minor issues in the review, I read the points covered again, and I believe that the revised version already addresses the issues highlighted in the review process. If publication is not possible, I would like to thank you for your time in proofreading.

RESPONSE TO REVIEWER 2:

1. **"The description of the clinical forms in lines 183-184 and Table 2".**

Thanks for the recommendation. We made the necessary adjustment.

2. **"The Abstract mentions "brown race" at 74% (this was not changed from the previous version) whereas in Table 1 it more correctly categorizes "Skin color – brown" at 72%".**

Thanks for the recommendation. We made the necessary adjustment.

3. **"The start of the paragraph "Discussion" begins abruptly with "This finding aligns…"**

Thanks for the recommendation. We made the adjustment at the beginning of the discussion.
"The reduction in the number of leprosy cases is something sought by several countries around the world. Our findings are in line with a study by Da Paz et al. (2022), which reported an 11,357 reduction in leprosy cases in Brazil during 2020, representing a 41% decrease compared to the average number of cases between 2015 and 2019."

4. **It is interesting that in Figure 2, two neighborhoods that appear on the map with the second highest risk (yellow), Perola and Maica, are shown as low risk on the spatial cluster map whereas Fatima in the north was calculated as having a high risk. Looks like the spatial clustering tool washed out the relatively high risk of these two neighborhoods.**

Thank you for your insightful observation regarding the relative risk levels presented in Figure 2. The difference you've noted between the individual neighborhood risk assessments and the spatial cluster map highlights an important aspect of the analysis: the spatial clustering tool aggregates data over larger areas, which can sometimes lead to a smoothing effect, or "washing out" of local variations.
To clarify, the relative risk scores for individual neighborhoods, such as Perola and Maica, are calculated using localized data specific to those neighborhoods. This can result in higher relative risk scores if there are concentrated factors or events that elevate the risk locally.
On the other hand, the spatial clustering tool considers not only the individual neighborhood data but also the surrounding context. It groups neighborhoods into clusters and calculates the risk based on the combined data. This method aims to identify broader areas of high or low risk, which may not reflect smaller-scale variations within the cluster. Thus, neighborhoods like Perola and Maica, while individually having higher relative risk scores, may be part of a larger cluster where the overall risk is lower when averaged with neighboring areas.

Furthermore, Fatima, despite being geographically distant from these neighborhoods, shows up as high risk in the spatial clustering map. This could be due to a number of reasons such as a higher incidence of the studied factor, a larger population at risk, or other regional influences that elevate the risk when viewed in the context of its cluster. We believe this explanation resolves the concern you have raised, and we will make sure to include a discussion of this methodological nuance in the revised manuscript to ensure clarity for all readers.

5. **Line 282, reference De Anjos et al. (2021) not found (Anjos et al.?)**

The reference is correct. Below is the article's citation data. Even so, we inserted the DOI.
ANJOS, L. H. G.; CUNHA, S. M. da; BATISTA, G. M.; HIGINO, T. M. M.; SOUZA, D. C. P. de.; ALIANÇA, A. S. dos S. Epidemiological profile of Leprosy in the state of Maranhão from 2018 to 2020. **Research, Society and Development**, *[S. l.]*, v. 10, n. 15, p. e272101523156, 2021. DOI: 10.33448/rsd-v10i15.23156. Disponível em: https://rsdjournal.org/index.php/rsd/article/view/23156.

RESPONSE TO REVIEWER 4:

1. **63: M. leprae and or M. Lepromatosis**

We appreciate the comments. We made the textual adjustment referring to the authors presented below.
Han XY, Seo YH, Sizer KC, Schoberle T, May GS, Spencer JS, Li W, Nair RG. A New *Mycobacterium* Species Causing Diffuse Lepromatous Leprosy, *American Journal of Clinical Pathology*, Volume 130, Issue 6, December 2008, Pages 856–864, https://doi.org/10.1309/AJCPP72FJZZRRVMM
Deps P, Collin SM. Mycobacterium lepromatosis as a Second Agent of Hansen´s Disease. Front Microbiol., 2021; 12. Sec Evolutionary and Genomic Microbiology. Doi: https://doi.org/10.3389/fmicb.2021.698588.

2. **64: in the skin also often Schwann cells particular Remak Schwann cells**.

We appreciate the comments. We made the textual adjustment referring to the author presented below.
Harty BL, Monk KR. Unwrapping the unappreciated: recent progress in Remak Schwann cell biology, Current Opinion in Neurobiology, Volume 47, 2017, Pages 131-137, ISSN 0959-4388, https://doi.org/10.1016/j.conb.2017.10.003.

3. **70: To be sure that it is leprosy you need 2 out of the 3 cardinal signs**.

We appreciate the comments. We made the textual adjustment referring to the author presented below.
Mennuru NB, Birudala R, Birudala G. Chapter 49 - Pathogenesis, clinical manifestations, and treatment of leprosy, Editor(s): Debasis Bagchi, Amitava Das, Bernard William Downs, Viral, Parasitic, Bacterial, and Fungal Infections, Academic Press, 2023, Pages 609-615, ISBN 9780323857307, https://doi.org/10.1016/B978-0-323-85730-7.00042-4.

4. **76: You can only speak of stopping the bacterial reproduction (cure may be only at the paucibacillary site) At the lepromatous site you have often a relapse (often not seen because the follow-up is to short) or reinfection.**

We appreciate the comments. We made the textual adjustment.

5. **84: There is still a lot of discussion about the 3 drugs for PB and only! year for MB particularly with a high BI. Or polar LL.**

We appreciate the comments. We highlight that the aforementioned issue is based on the WHO, 2019 publication.

6. **92: Not only of general public but of health professionals too.**

We appreciate the comments. We made the textual adjustment.

7. **104: I noticed no PREP. I fully agree. May be a place here for you to explain the reader why?**

We appreciate the comments. We made the textual adjustment.
Regarding the treatment of leprosy patients co-infected with HIV, studies highlight complexities and challenges. It was observed that infection with this virus does not seem to change the incidence or clinical spectrum of the disease, and does not cause changes, or does so discreetly, in the course of leprosy. (Cavalcante et al., 2021; Bezerra et al., 2021). Therefore, the symptoms, reactions and impact of ART and PREP are uncertain in cases of HIV/Leprosy, making it difficult for the patient to adhere to available treatment (Maia, 2019).

8. **112: I see here that you also will focus on healthcare workers. But it is also essential to have a direct supporting team by WhatsApp or internet where they can get support.**

We appreciate the comments. We made the textual adjustment.
Furthermore, professionals' access to technological resources for monitoring cases, encouraging diagnoses, such as bacilloscopy, should be expanded in areas with a high cumulative incidence of leprosy, ensuring reliable diagnosis and adequate classification of patients as paucibacillary or multibacillary

9. **139: Do not use race but background African, Indian, Asian, chinees, from India, or European. Or mixed**

We appreciate the comments. We made the textual adjustment.

10. **In table 1 I seen you have used skin colour. And Indigenous. Mixed could also be important.**

We appreciate the comments, but we have entered the classification as shown in the Health Department's notification system

11. **In table 2 I miss the disabilities and the delay in diagnosis. These are very important to plan your extra focus points. And are more important than prevalence. And may help you to understand the epidemiology in that area.**

We appreciate the comments. No data on disabilities were provided and this justifies not including them in table 2. Furthermore, we included in the discussion the impact caused by the delay in diagnosing patients with leprosy.

12. **280: Look not only at skin colour but at poverty too, that may be more important. I see you mention it in 285-288 and thereafter.**

Thank you for your observation. We highlight that poverty was mentioned in later lines, as the text developed.

13. **I wonder whether this specific research will contribute to the knowledge of the reader. Because all was known already**

This study presents a relevant, pertinent and necessary theme as it concerns a Neglected Disease and the addition of the distribution of cases over the period 2011-2020 in Santarém through GIS mapping and calculation of relative risk (RR) in neighborhoods using spatial epidemiological tools, will certainly be different for our city and country. It will enable an important presentation and subsequent intervention by managers and health professionals.

RESPONSE TO REVIEWER 5:

1. **Lines 30 and 63 use italics for *Mycobacterium leprae***

We appreciate the comments. We made the textual adjustment.

2. **The majority of the references in the introduction are from authors from Brazil, more diverse references are needed to describe the pathology and clinical signs of the disease.**

We appreciate the comments. We made the textual adjustment and inserted the references presented below.

Ebenezer GJ, Scollard DM. Treatment and evaluation advances in leprosy neuropathy. Neurotherapeutics 2021; 18:2337-50. DOI: https://doi.org/10.1007/s13311-021-01153-z.

Mi Z, Liu H, Zhang F. Advances in the immunology and genetics of leprosy. Front Immunol 2020; 11:567. DOI: https://doi.org/10.3389/fimmu.2020.00567.

3. **Lines 183 and 184 DL, VL, IL abs TL need to be spelled out.**

We appreciate the comments. We made the textual adjustment.

4. **Table 2: add IL, TL, VL and DL by the different forms of disease.**

We appreciate the comments. We made the textual adjustment.

5. **Figure 1: Needs caption and the colors of the variables are similar and cannot be differentiate.**

Figure 1 was removed due to lack of clarity in color identification.

6. **Line 246: Start the sentence at "It was observed", delete "In table 1 of the present study"**

We appreciate the comments. We made the textual adjustment.

7. **Lines 303-304: delete the percentages (73, 21, 4 and 2).**

We appreciate the comments. We made the textual adjustment.

8. **Line 319: delete "as demonstrated in the table 2"**

We appreciate the comments. We made the textual adjustment.

9. **Line 323: delete “shown in table 2”**
   We appreciate the comments. We made the textual adjustment.
10. **Line 347: delete “as shown in Table 2”**

We appreciate the comments. We made the textual adjustment.

11. **Lines 350-351: the statement “even though.. “is not clear just state that 31% of patients have nerve damage.**

We appreciate the comments. We made the textual adjustment.
