# Supplementary material for: Epidemiological, clinical, and geographical characterization of Leprosy in the County of Santarém-Pará: Insights for effective control and targeted intervention
Source: PLoS Negl Trop Dis. 2024 Mar 20;18(3):e0012063. doi: 10.1371/journal.pntd.0012063 (PMC10984465; doi:10.1371/journal.pntd.0012063)
Supplement: Attachment — Submitted filename: PLOS review February 2024.docx [file pntd.0012063.s003.docx]

Summary of the research

The authors of this manuscript described the epidemiological, clinical and geographical Characterization of leprosy in the Municipality of Santarem-Para. The authors stated that the results of this study might support health managers and professionals in developing public policies that will achieve prevention and early treatment in this endemic region of Brazil.

Minor issues

Lines 30 and 63 use italics for *Mycobacterium leprae*

Introduction

The majority of the references in the introduction are from authors from Brazil, more diverse references are needed to describe the pathology and clinical signs of the disease.

Methodology is adequate

Results

Lines 183 and 184 DL, VL, IL abs TL need to be spelled out.

Table 2: add IL, TL, VL and DL by the different forms of disease.

Figure 1: Needs caption and the colors of the variables are similar and cannot be differentiate.

Discussion

Line 246: Start the sentence at “It was observed”, delete “In table 1 of the present study”

Lines 303-304: delete the percentages (73, 21, 4 and 2).

Line 319: delete “as demonstrated in the table 2”

Line 323: delete “shown in table 2”

Line 347: delete “as shown in Table 2”

Lines 350-351: the statement “even though.. “is not clear just state that 31% of patients have nerve damage.
